# Supplementary material for: Input-and cell-type-specific developmental alterations to thalamic synapses in a Dravet syndrome mouse model
Source: bioRxiv. 2026 Feb 13:2026.02.12.705567. Preprint. [Version 1] doi: 10.64898/2026.02.12.705567 (PMC12918865; doi:10.64898/2026.02.12.705567)
Supplement: Supplement 1 [file media-1.pdf]

Supplemental Material

Input-and cell-type-specific developmental alterations to thalamic synapses in a Dravet syndrome mouse model

Mona Safari[1,2], Rutvi Desai[1], Harbal Rai[1], Tyler J. Roberts[1], Rabeya Khondaker[1,2], Julia Smith[1], Sharon A. Swanger[1,3,4]

[1]Fralin Biomedical Research Institute at VTC, Virginia Tech, Roanoke, VA

[2]Translational Biology, Medicine, and Health Graduate Program, Virginia Tech, Blacksburg, VA

[3]Department of Biomedical Sciences and Pathobiology, VA-MD College of Veterinary Medicine, Virginia Tech, Blacksburg, VA

[4]Department of Internal Medicine, VTC School of Medicine, Roanoke, VA

Corresponding Author:

Sharon A. Swanger

Fralin Biomedical Research Institute at VTC

Center for Neurobiology Research

2 Riverside Circle

Roanoke, VA 24016

saswanger@vtc.vt.edu

Contents:

Supplemental Figures S1 – S8

Supplemental Tables S1 – S4

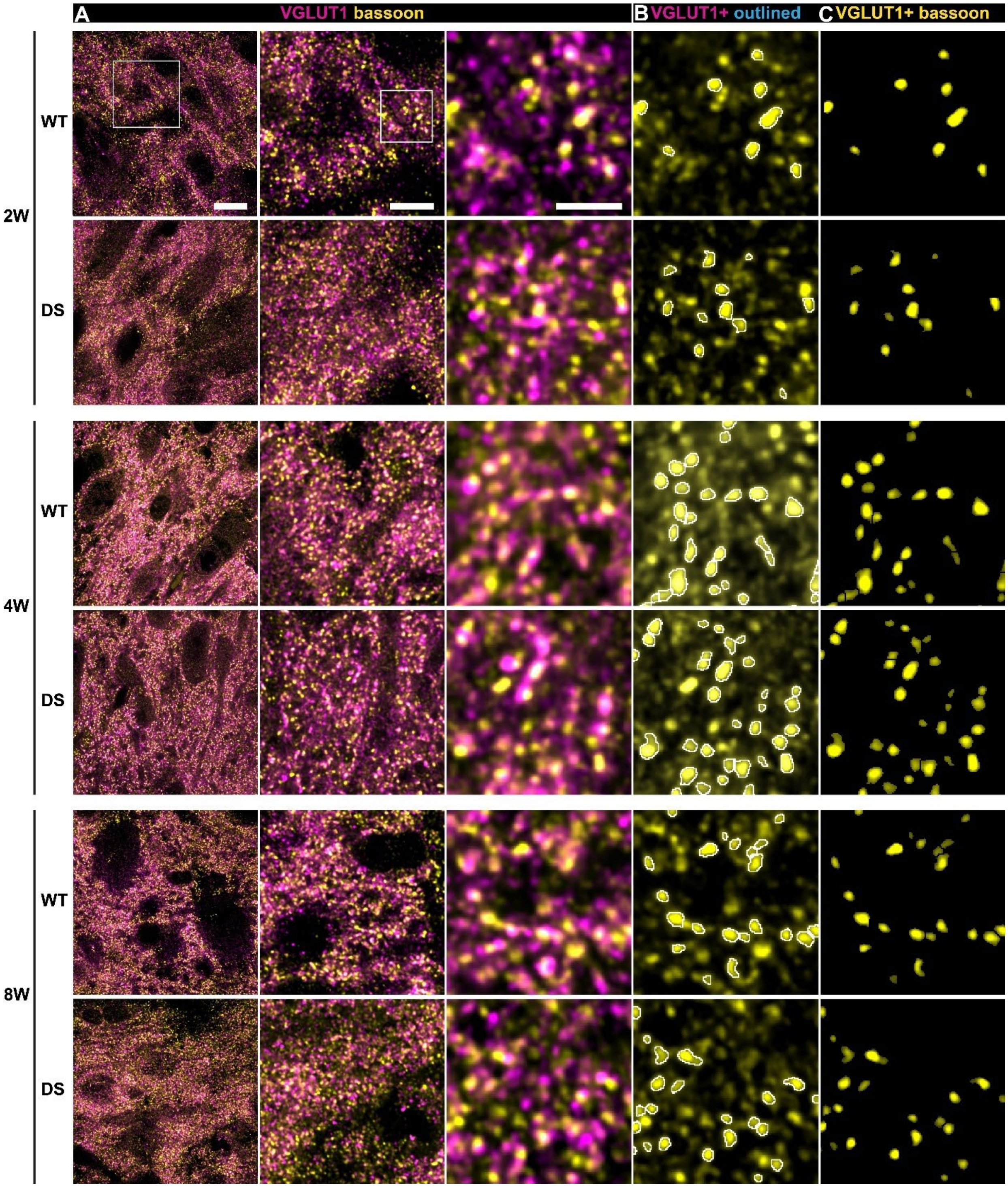

Figure S1. Raw images for VGLUT1-positive synapse quantification in the VPL.

A. 60x images with 4x SoRa of VGLUT1 and bassoon immunostained brain sections from WT and DS mice aged 2W, 4W, and 8W are shown. Regions of the full field image (left) were expanded to show synapse density (middle) and individual synapses (right). Expanded regions in subsequent rows are the same size. Scales: 10 µm, 5 µm, and 2 µm.

B. Image shows bassoon signal from the image in panel A with VGLUT1-positive puncta outlined (white).

C. Image shows only VGLUT1-positive bassoon puncta, which was overlaid with VGLUT1 in Figure 3.

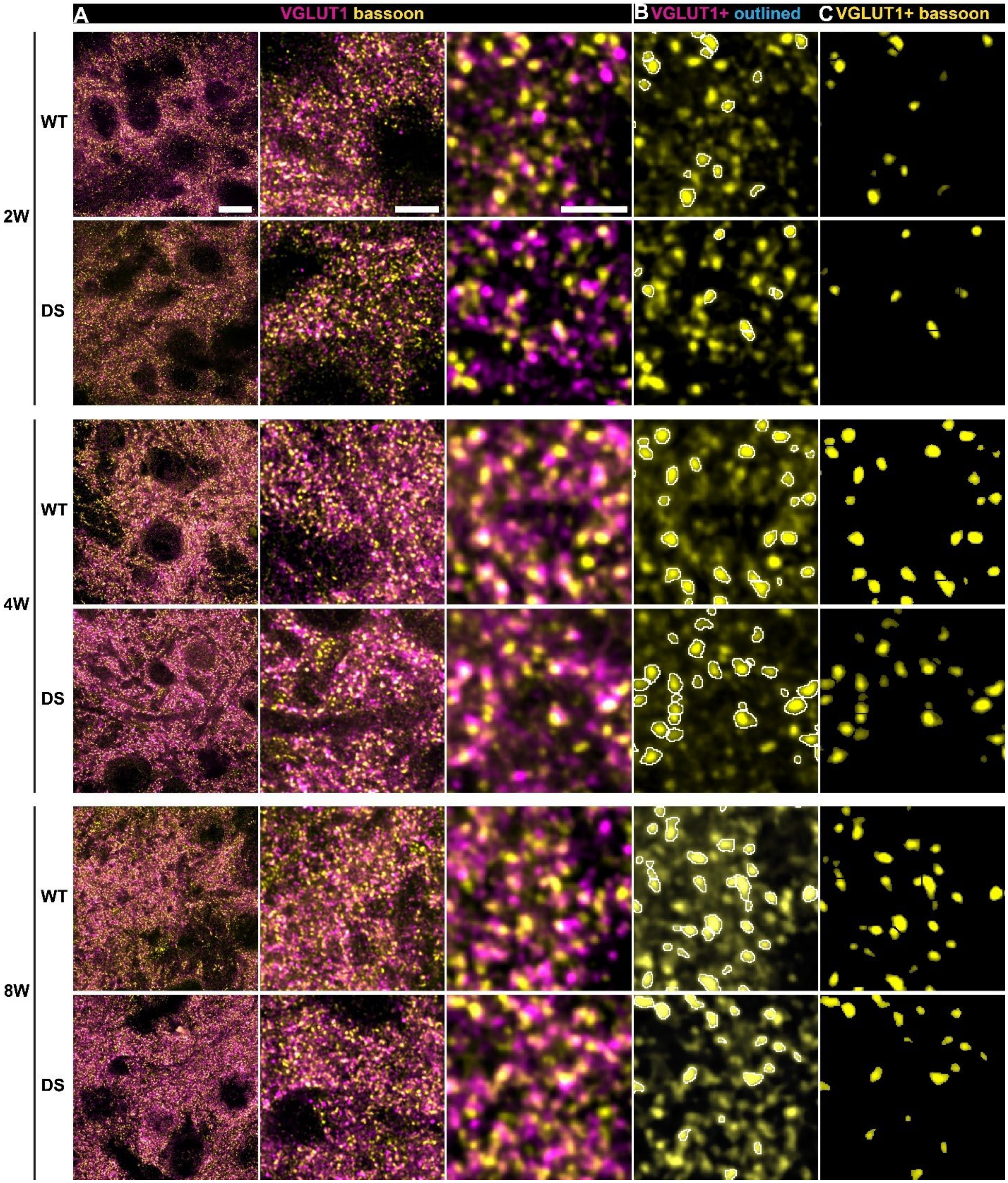

Figure S2. Raw images for VGLUT1-positive synapse quantification in the VPM.

A. 60x images with 4x SoRa of VGLUT1 and bassoon immunostained brain sections from WT and DS mice aged 2W, 4W, and 8W are shown. Regions of the full field image (left) were expanded to show synapse density (middle) and individual synapses (right). Expanded regions in subsequent rows are the same size. Scales: 10 µm, 5 µm, and 2 µm.

B. Image shows bassoon signal from the image in panel A with VGLUT1-positive puncta outlined (white).

C. Image shows only VGLUT1-positive bassoon puncta, which was overlaid with VGLUT1 in Figure 3.

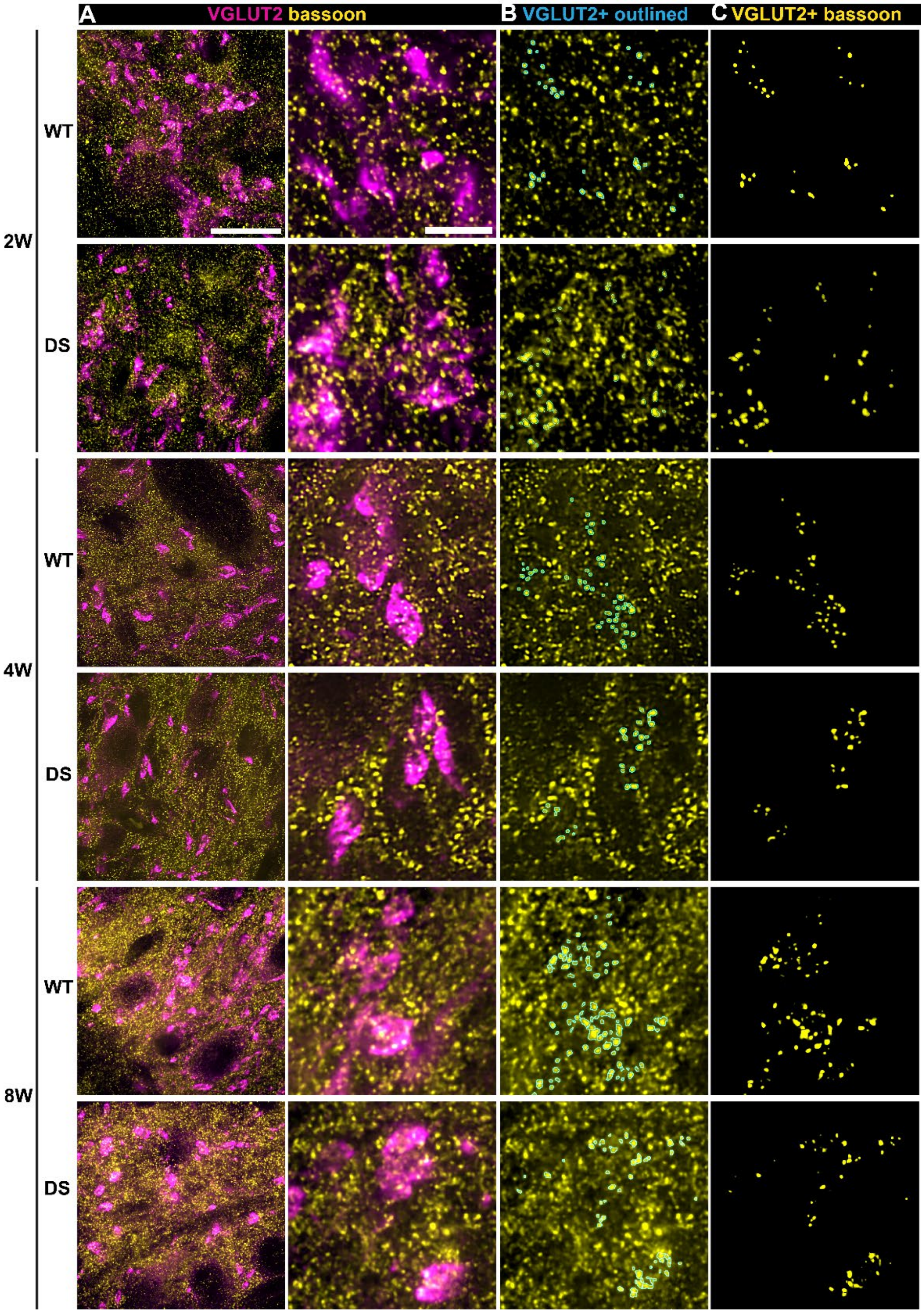

Figure S3. Raw images for VGLUT2-positive synapse quantification in the VPL.

A. 60x images with 4x SoRa of VGLUT2 and bassoon immunostained brain sections from WT and DS mice aged 2W, 4W, and 8W are shown. Regions of the full field image (left) were expanded to show axon terminals with multiple bassoon puncta (right). Expanded regions in subsequent rows are the same size. Scales: 10 µm and 2 µm.

B. Image shows bassoon signal from the image in panel A with VGLUT2-positive puncta outlined (cyan).

C. Image shows only VGLUT2-positive bassoon puncta, which was overlaid with VGLUT2 in Figure 4.

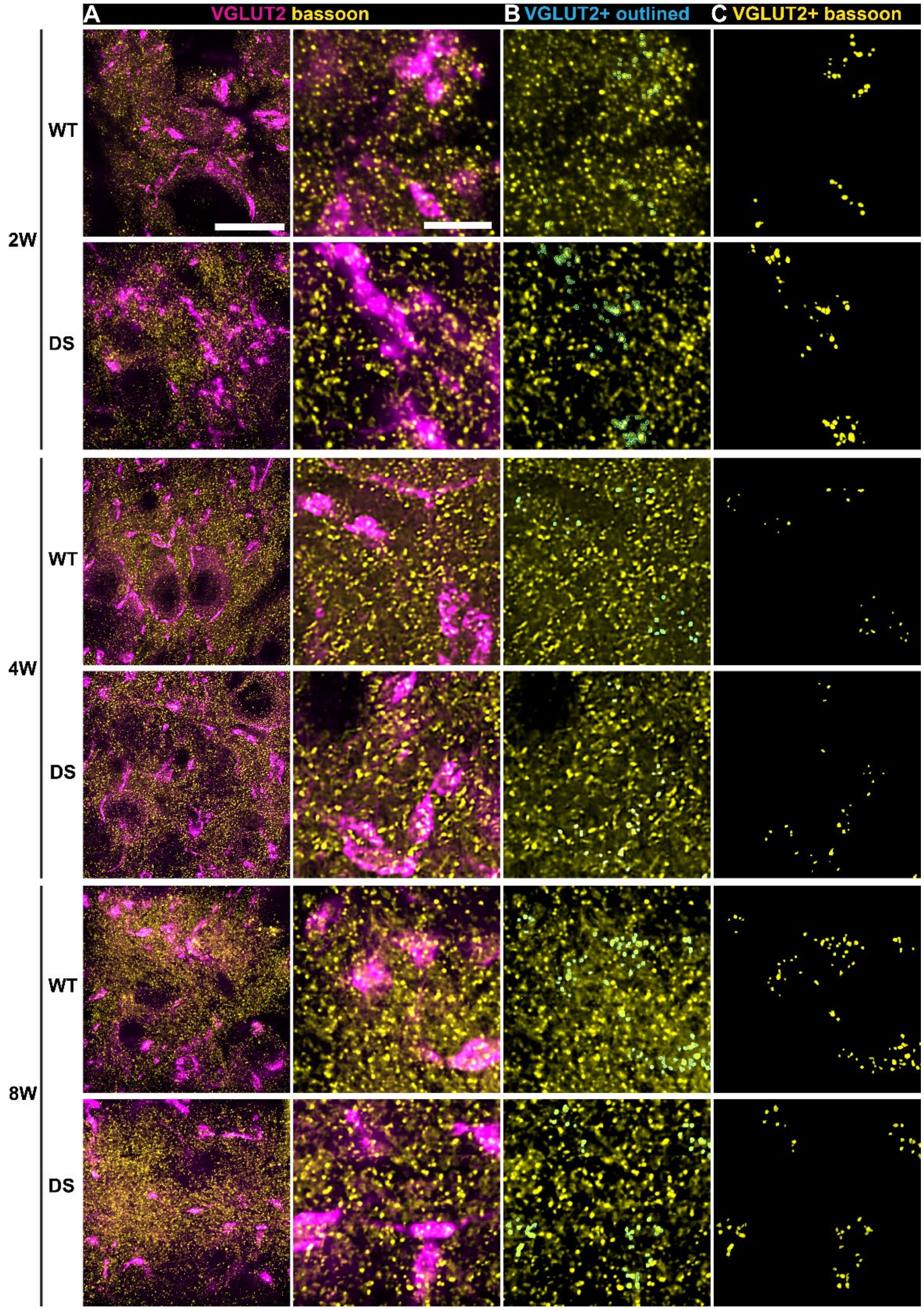

Figure S4. Raw images for VGLUT2-positive synapse quantification in the VPM.

A. 60x images with 4x SoRa of VGLUT2 and bassoon immunostained brain sections from WT and DS mice aged 2W, 4W, and 8W are shown. Regions of the full field image (left) were expanded to show axon terminal with multiple bassoon puncta (right). Expanded regions in subsequent rows are the same size. Scales: 10 µm and 2 µm.

B. Image shows bassoon signal from the image in panel A with VGLUT2-positive puncta outlined (cyan).

C. Image shows only VGLUT2-positive bassoon puncta, which was overlaid with VGLUT2 in Figure 4.

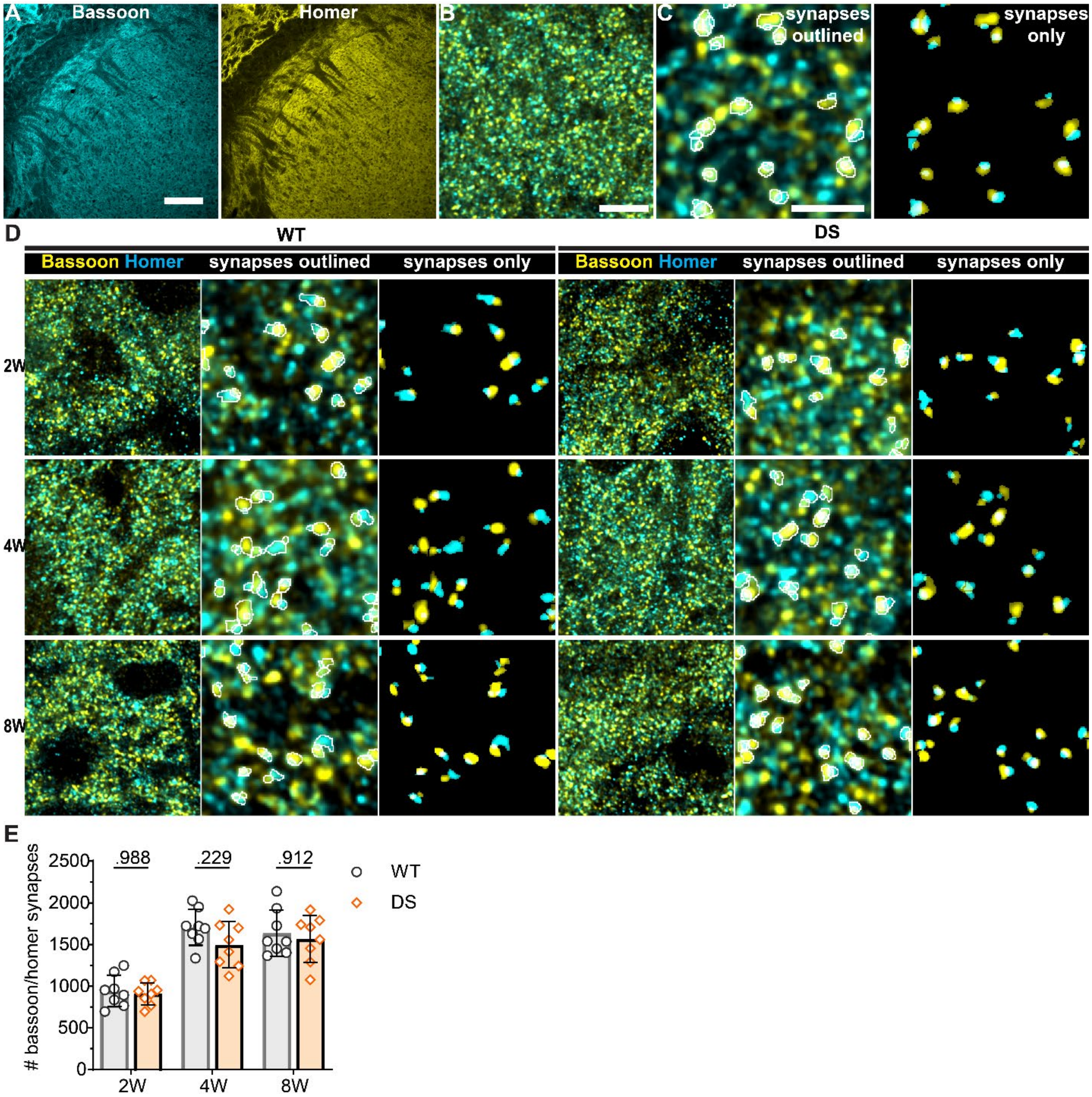

Figure S5. Total VPL glutamatergic synapse number is similar between WT and DS mice.

A. Representative 10x and **(B)** 60x (4x SoRa) images show homer and bassoon immunostaining of the thalamus. Scale: 200 µm and 10 µm.

C. Expanded region from the 60x (4x SoRa) image shows overlapping homer and bassoon puncta outlined (white, left) and only the puncta identified as synapses shown at right. Scale: 2 µm.

D. Representative ROIs from 60x images show homer and bassoon immunostained brain sections from WT and DS mice aged 2W (WT: n = 8, DS: n = 8), 4W (WT: n = 8, DS: n = 8), and 8W (WT: n = 8, DS: n = 8). Expanded images show overlapping pairs (synapses) outlined and synaptic puncta only (at right).

E. The number of synapses were averaged across three images from the VPL in each mouse. The group mean and SD were plotted with data points for all mice. Data were analyzed by one-way ANOVA with posthoc Sidak's tests between genotypes at each age (p-values shown in plots).

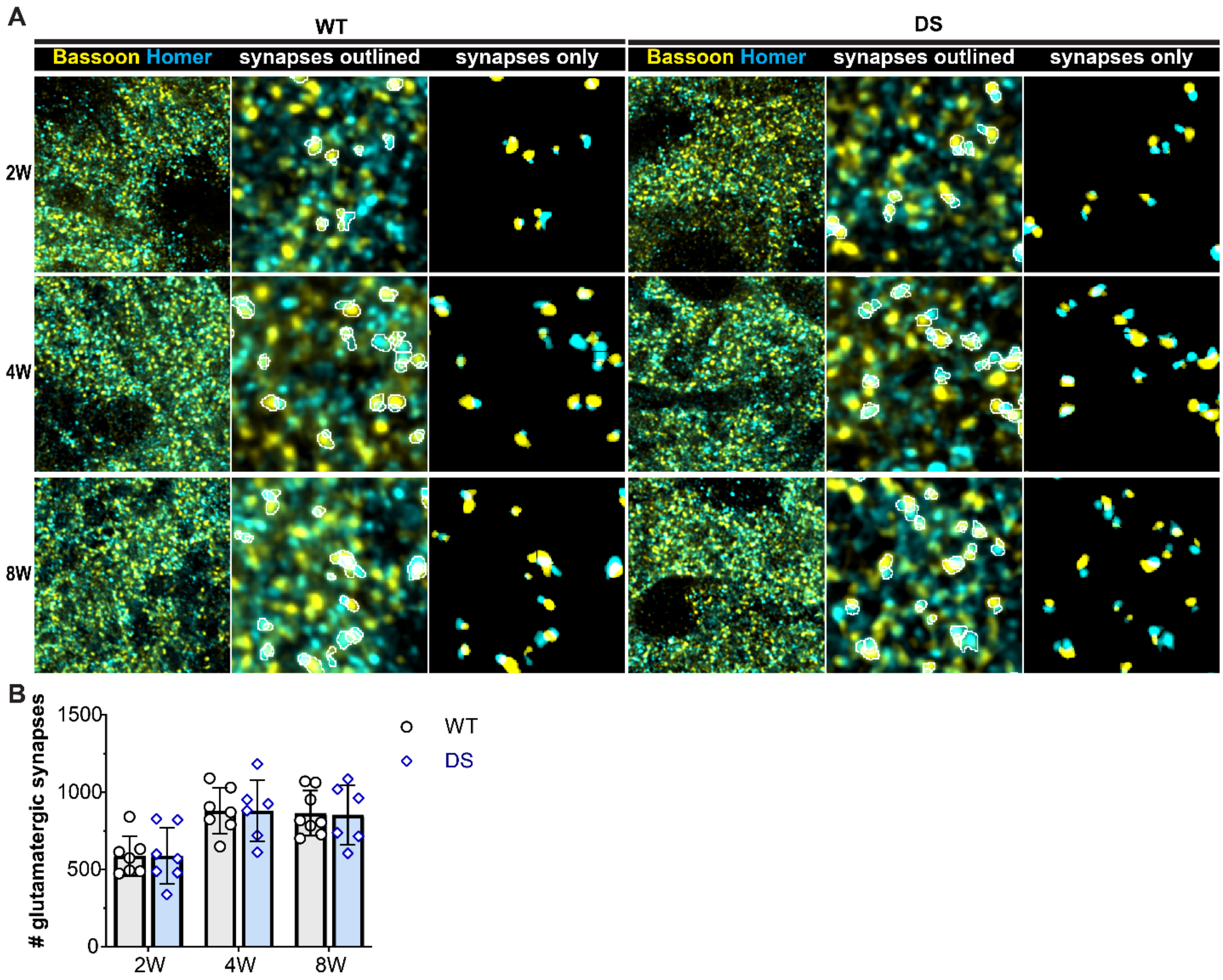

Figure S6. Total VPM glutamatergic synapse number is similar between WT and DS mice.

A. Representative 10x and **(B)** 60x (4x SoRa) images show homer and bassoon immunostaining of the thalamus. Scale: 200 µm and 10 µm.

B. Expanded region from the 60x (4x SoRa) image shows overlapping homer and bassoon puncta outlined (white, left) and only the puncta identified as synapses shown at right. Scale: 2 µm.

C. Representative ROIs from 60x images show homer and bassoon immunostained brain sections from WT and DS mice aged 2W (WT: n = 8, DS: n = 8), 4W (WT: n = 8, DS: n = 8), and 8W (WT: n = 8, DS: n = 8). Expanded images show overlapping pairs (synapses) outlined and synaptic puncta only (at right).

D. The number of synapses were averaged across three images from the VPM in each mouse. The group mean and SD were plotted with data points for all mice. Data were analyzed by one-way ANOVA with posthoc Sidak's tests between genotypes at each age (p-values shown in plots).

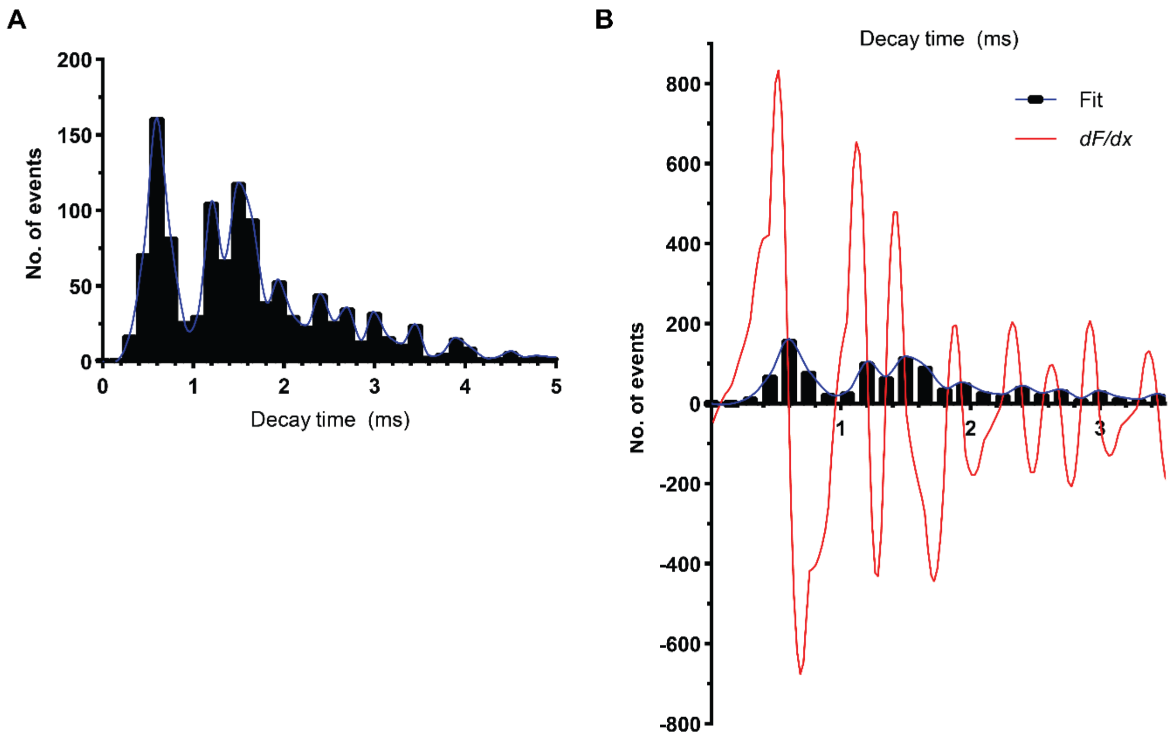

Figure S7. Representative histograms for establishing Type 1 and Type 2 mEPSCs.

A. Histogram shows total number of events vs decay time, with events binned across 0.2 ms. Data were interpoplated with a non-smoothing spline (blue line).

B. The derivative (*dF/dx,* red line) of the spline was plotted, and the decay time value where the *dF/dx* plot crosses zero between the two prominent histogram peaks was used at the cutoff value for Type 1 and Type 2 mEPSCs.

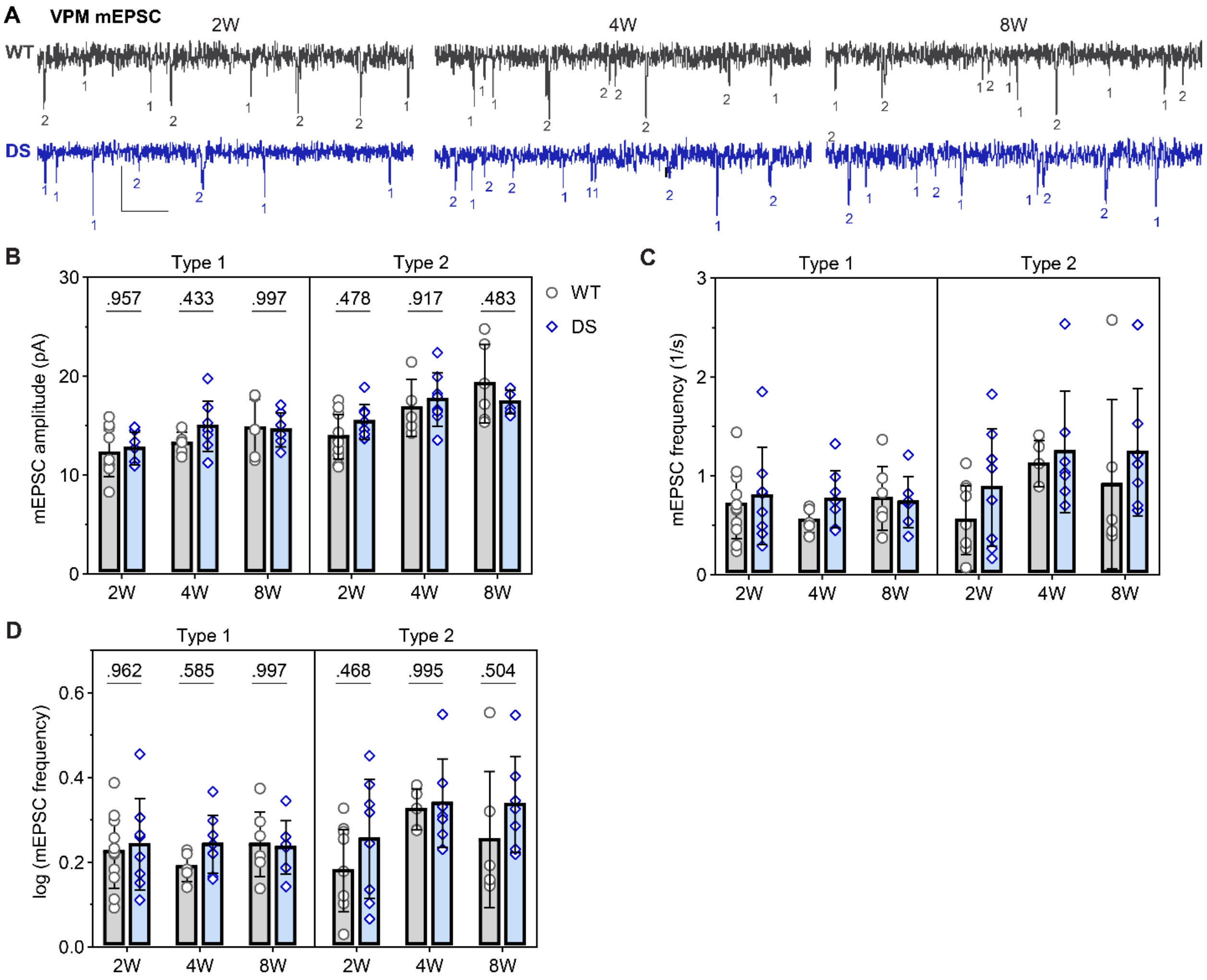

Figure S8. The frequency and amplitude of VPM Type 1 and 2 mEPSC are similar between WT and DS mice.

A. Representative voltage-clamp recordings from VPM neurons show mEPSCs in WT and DS mice aged P13 – P17 (WT: n = 11; DS: n = 7), P28 – P32 (WT: n = 7; DS: n = 8), P57 – P62 (WT: n = 7; DS: n = 7) with each mEPSC denoted as Type 1 (fast, putative sensory) or Type 2 (slow, putative CT) based on their decay kinetics.

B. Mean mEPSC amplitude were plotted for each mouse with the group mean ± *SD*. The data were analyzed by two-way ANOVA with pairwise Sidak's comparisons (p-values shown on plots) between WT and DS at each age.

C. The mean mEPSC frequency was plotted as raw data (geometric mean ± 95CI) and testing showed a non-normal distribution.

D. Transformed log10 values (mean ± 95CI) of the mean mESPC frequency were plotted and analyzed by two-way ANOVA with pairwise Sidak's comparisons (p-values shown on plots) between WT and DS at each age.

Supplemental Table 1. Summary of electrophysiology normality and heteroscedasticity statistics.

	D'Agostino-Pearson test for Normality of residuals				Spearman's test for heteroscedasticity			
	Raw Data		Log transformed data		Raw Data		Log transformed data	
**EPSC Frequency**	K2	p-value	K2	p-value	R	p-value	R	p-value
VPL sEPSC	18.00	<.001	0.79	0.091	0.244	0.039	0.039	0.889
VPM sEPSC	14.23	0.001	3.33	0.190	0.539	<.001	0.313	0.014
VPL mEPSC	19.37	<.001	1.61	0.447	0.449	<.001	0.043	0.382
VPM mEPSC	30.78	<.001	0.96	0.081	0.169	0.128	0.540	0.358
VPL mEPSC Type 1	23.43	<.001	5.39	0.068	0.461	<.001	0.247	0.051
VPL mEPSC Type 2	17.57	<.001	3.88	0.144	0.333	0.010	0.189	0.099
VPM mEPSC Type 1	12.59	0.002	3.69	0.158	0.193	0.099	0.102	0.251
VPM mEPSC Type 2	14.29	0.001	4.01	0.134	-0.031	0.421	-0.213	0.085
**EPSC Amplitude**	K2	p-value			R	p-value		
VPL sEPSC	4.29	0.117	-	-	0.088	0.262	-	-
VPM sEPSC	3.43	0.180	-	-	0.158	0.134	-	-
VPL mEPSC	1.68	0.431	-	-	0.030	0.409	-	-
VPM mEPSC	2.73	0.255	-	-	0.028	0.423	-	-
VPL mEPSC Type 1	1.05	0.592	-	-	0.228	0.066	-	-
VPL mEPSC Type 2	2.99	0.224	-	-	0.099	0.254	-	-
VPM mEPSC Type 1	1.03	0.597	-	-	-0.049	0.377	-	-
VPM mEPSC Type 2	2.49	0.288	-	-	0.172	0.130	-	-
**IPSC Frequency**	K2	p-value	K2	p-value	R	p-value	R	p-value
VPL sIPSC	10.37	0.006	1.91	0.385	0.328	0.006	-0.024	0.431
VPM sIPSC	7.95	0.019	1.09	0.579	0.322	0.009	0.034	0.404
VPL mIPSC	7.87	0.020	0.24	0.885	0.282	0.012	-0.023	0.428
VPM mIPSC	7.48	0.024	1.02	0.601	0.462	<.001	0.254	0.033
**IPSC Amplitude**	K2	p-value			K2	p-value		
VPL sIPSC	5.14	0.077	-	-	-0.114	0.202	-	-
VPM sIPSC	1.43	0.489	-	-	-0.090	0.264	-	-
VPL mIPSC	2.81	0.245	-	-	0.105	0.199	-	-
VPM mIPSC	1.06	0.590	-	-	0.256	0.024	-	-

Supplemental Table 2. Summary of electrophysiology data.

	Frequency												Amplitude					
	Raw data: Geometric mean [95 CI]						Transformed data: Mean [95 CI]						Raw data: Mean (*SD*)					
	WT			DS			WT			DS			WT			DS		
**VPL sEPSC**	GM	UL	LL	GM	UL	LL	M	UL	LL	M	UL	LL	M	SD	n	M	SD	n
2W	3.05	4.05	2.30	3.22	4.99	2.07	0.48	0.61	0.36	0.51	0.70	0.32	16.89	2.60	10	16.20	0.91	6
4W	3.59	4.85	2.66	2.11	2.87	1.55	0.55	0.69	0.42	0.32	0.46	0.19	17.70	4.30	11	15.03	3.49	9
8W	3.82	5.35	2.73	2.50	3.87	1.62	0.58	0.73	0.44	0.40	0.59	0.21	15.91	2.62	11	17.35	2.97	8
**VPM sEPSC**	GM	UL	LL	GM	UL	LL	M	UL	LL	M	UL	LL	M	SD	n	M	SD	n
2W	1.55	1.93	1.24	1.57	2.21	1.11	0.41	0.47	0.35	0.42	0.51	0.32	14.21	1.45	9	14.03	2.32	8
4W	2.30	3.40	1.56	2.23	2.87	1.73	0.53	0.65	0.41	0.51	0.59	0.44	17.35	4.15	9	16.03	2.86	11
8W	2.13	2.97	1.52	2.28	3.59	1.45	0.50	0.60	0.40	0.53	0.66	0.39	16.72	2.66	7	17.30	2.55	7
**VPL mEPSC**	GM	UL	LL	GM	UL	LL	M	UL	LL	M	UL	LL	M	SD	n	M	SD	n
2W	3.34	4.84	2.30	3.27	4.26	2.51	0.52	0.68	0.36	0.51	0.63	0.40	12.14	1.80	9	13.12	1.87	6
4W	3.52	4.42	2.80	1.84	2.30	1.46	0.55	0.65	0.45	0.26	0.36	0.17	17.70	1.55	8	14.28	1.93	9
8W	3.23	4.91	2.13	2.00	2.55	1.57	0.51	0.69	0.33	0.30	0.41	0.19	17.12	2.83	8	18.58	2.86	10
**VPM mEPSC**	GM	UL	LL	GM	UL	LL	M	UL	LL	M	UL	LL	M	SD	n	M	SD	n
2W	1.60	2.23	1.15	1.90	2.80	1.29	0.20	0.35	0.06	0.28	0.45	0.11	12.50	2.25	11	14.07	1.80	8
4W	1.83	2.65	1.27	1.82	2.43	1.36	0.26	0.42	0.10	0.26	0.39	0.13	13.93	2.60	7	13.10	1.98	8
8W	2.12	3.07	1.47	2.43	3.53	1.67	0.33	0.49	0.17	0.39	0.55	0.22	13.90	2.87	7	12.88	2.00	9
**VPL T1 mEPSC**	GM	UL	LL	GM	UL	LL	M	UL	LL	M	UL	LL	M	SD	n	M	SD	n
2W	1.16	1.59	0.84	1.02	1.28	0.82	0.34	0.41	0.26	0.31	0.36	0.26	12.36	1.33	6	11.71	1.37	9
4W	1.94	2.57	1.47	0.76	0.93	0.62	0.47	0.56	0.39	0.25	0.29	0.21	16.07	2.53	8	13.45	1.52	8
8W	1.61	2.87	0.91	1.06	1.44	0.78	0.43	0.58	0.29	0.32	0.39	0.25	14.77	2.13	7	16.40	2.13	8
**VPM T1 mEPSC**	GM	UL	LL	GM	UL	LL	M	UL	LL	M	UL	LL	M	SD	n	M	SD	n
2W	0.63	0.91	0.44	0.69	1.11	0.42	0.23	0.28	0.17	0.24	0.33	0.15	12.20	2.34	11	12.68	1.65	7
4W	0.54	0.73	0.40	0.72	0.98	0.53	0.19	0.23	0.15	0.24	0.30	0.19	13.20	1.15	7	14.93	2.54	8
8W	0.72	1.05	0.49	0.70	0.97	0.50	0.24	0.31	0.17	0.24	0.29	0.18	14.77	2.85	7	14.56	1.71	7
**VPL T2 mEPSC**	GM	UL	LL	GM	UL	LL	M	UL	LL	M	UL	LL	M	SD	n	M	SD	n
2W	1.81	2.69	1.21	1.80	2.44	1.33	1.36	1.63	1.10	1.37	1.59	1.14	14.15	2.47	6	12.85	1.56	9
4W	1.68	2.19	1.29	1.19	1.66	0.85	1.31	1.48	1.14	1.11	1.29	0.92	17.11	2.40	8	16.10	2.12	7
8W	1.52	2.45	0.95	1.41	1.72	1.16	1.28	1.56	1.01	1.20	1.32	1.07	16.89	2.90	9	18.70	2.49	8
**VPM T2 mEPSC**	GM	UL	LL	GM	UL	LL	M	UL	LL	M	UL	LL	M	SD	n	M	SD	n
2W	0.43	0.78	0.24	0.67	1.38	0.32	0.18	0.25	0.11	0.25	0.37	0.14	13.87	2.26	11	15.40	1.78	8
4W	1.10	1.43	0.85	1.15	1.69	0.78	0.32	0.38	0.26	0.34	0.44	0.24	16.81	2.87	5	17.64	2.69	8
8W	0.70	1.52	0.32	1.12	1.73	0.73	0.25	0.42	0.08	0.34	0.44	0.23	19.24	3.98	6	17.41	1.18	7
**VPL sIPSC**	GM	UL	LL	GM	UL	LL	M	UL	LL	M	UL	LL	M	SD	n	M	SD	n
2W	6.14	8.81	4.28	7.32	9.96	5.38	0.79	0.94	0.63	0.86	1.00	0.73	25.31	4.33	11	25.23	5.74	9
4W	7.29	10.13	5.24	3.66	5.18	2.58	0.86	1.01	0.72	0.56	0.71	0.41	25.84	5.42	9	26.83	6.25	8
8W	8.22	11.37	5.95	4.81	6.39	3.63	0.92	1.06	0.77	0.68	0.81	0.56	29.90	4.29	8	27.34	2.98	11
**VPM sIPSC**	GM	UL	LL	GM	UL	LL	M	UL	LL	M	UL	LL	M	SD	n	M	SD	n
2W	8.69	10.98	6.88	6.55	10.28	4.18	0.94	1.04	0.84	0.82	1.01	0.62	27.11	6.58	10	22.13	2.57	9
4W	9.19	13.31	6.35	4.14	5.99	2.86	0.96	1.12	0.80	0.62	0.78	0.46	28.47	6.24	11	26.07	4.56	9
8W	7.45	11.35	4.88	6.57	9.24	4.67	0.87	1.06	0.69	0.82	0.97	0.67	28.78	1.58	7	28.68	7.74	6
**VPL mIPSC**	GM	UL	LL	GM	UL	LL	M	UL	LL	M	UL	LL	M	SD	n	M	SD	n
2W	6.07	8.95	4.11	5.53	7.68	3.98	0.78	0.95	0.61	0.74	0.89	0.60	23.20	3.61	12	21.97	2.13	10
4W	7.79	10.19	5.96	4.03	5.32	3.06	0.89	1.01	0.78	0.61	0.73	0.49	26.90	3.92	12	25.28	5.42	13
8W	5.36	7.36	3.91	3.37	4.82	2.36	0.73	0.87	0.59	0.53	0.68	0.37	25.63	3.92	10	25.32	3.20	9
**VPM mIPSC**	GM	UL	LL	GM	UL	LL	M	UL	LL	M	UL	LL	M	SD	n	M	SD	n
2W	4.72	6.54	3.40	6.25	9.33	4.18	0.67	0.82	0.53	0.80	0.97	0.62	21.29	3.18	11	21.46	2.83	10
4W	4.85	6.53	3.60	6.24	10.34	3.76	0.69	0.81	0.56	0.80	1.01	0.58	22.77	3.71	11	25.14	4.81	10
8W	3.71	4.40	3.13	4.84	6.89	3.40	0.57	0.64	0.50	0.69	0.84	0.53	27.33	4.06	8	28.25	5.51	10

Supplemental Table 3. Electrophysiology data ANOVA tables.

	Frequency ANOVA table			Amplitude ANOVA table		
**VPL sEPSC**	ANOVA table	F (DFn, DFd)	P value	ANOVA table	F (DFn, DFd)	P value
2W	Interaction	F (2, 47) = 1.99	P=.148	Interaction	F (2, 49) = 2.064	P=.138
4W	Age	F (2, 47) = 0.481	P=.621	Row Factor	F (2, 49) = 0.036	P=.964
8W	Genotype	F (1, 47) = 5.98	P=.018	Column Factor	F (1, 49) = 0.556	P=.459
**VPM sEPSC**	ANOVA table	F (DFn, DFd)	P value	ANOVA table	F (DFn, DFd)	P value
2W	Interaction	F (2, 44) = 0.114	P=.893	Interaction	F (2, 45) = 0.140	P=.869
4W	Row Factor	F (2, 44) = 4.199	P=.021	Row Factor	F (2, 45) = 5.465	P=.008
8W	Column Factor	F (1, 44) = 0.026	P=.872	Column Factor	F (1, 45) < 0.001	P=.976
**VPL mEPSC**	ANOVA table	F (DFn, DFd)	P value	ANOVA table	F (DFn, DFd)	P value
2W	Interaction	F (2, 46) = 3.14	P=.052	Interaction	F (2, 45) = 6.354	P=.004
4W	Row Factor	F (2, 46) = 2.80	P=.071	Row Factor	F (2, 45) = 22.15	P<.001
8W	Column Factor	F (1, 46) = 12.8	P<.001	Column Factor	F (1, 45) = 0.264	P=.610
**VPM mEPSC**	ANOVA table	F (DFn, DFd)	P value	ANOVA table	F (DFn, DFd)	P value
2W	Interaction	F (2, 41) = 0.205	P=.794	Interaction	F (2, 44) = 1.780	P=.181
4W	Row Factor	F (2, 41) = 0.217	P=.082	Row Factor	F (2, 44) = 0.042	P=.958
8W	Column Factor	F (1, 41) = 0.430	P=.664	Column Factor	F (1, 44) = 0.021	P=.885
**VPL T1 mEPSC**	ANOVA table	F (DFn, DFd)	P value	ANOVA table	F (DFn, DFd)	P value
2W	Interaction	F (2, 39) = 4.356	P=.020	Interaction	F (2, 40) = 4.840	P=.013
4W	Row Factor	F (2, 39) = 1.328	P=.277	Row Factor	F (2, 40) = 14.05	P<.001
8W	Column Factor	F (1, 39) = 20.38	P<.001	Column Factor	F (1, 40) = 0.9523	P=.335
**VPM T1 mEPSC**	ANOVA table	F (DFn, DFd)	P value	ANOVA table	F (DFn, DFd)	P value
2W	Interaction	F (2, 39) = 5.644	P=.007	Interaction	F (2, 38) = 0.641	P=.533
4W	Row Factor	F (2, 39) = 2.764	P=.075	Row Factor	F (2, 38) = 4.245	P=.022
8W	Column Factor	F (1, 39) = 31.01	P<.001	Column Factor	F (1, 38) = 0.972	P=.331
**VPL T2 mEPSC**	ANOVA table	F (DFn, DFd)	P value	ANOVA table	F (DFn, DFd)	P value
2W	Interaction	F (2, 42) = 0.626	P=.540	Interaction	F (2, 41) = 2.127	P=.132
4W	Row Factor	F (2, 42) = 1.568	P=.221	Row Factor	F (2, 41) = 13.48	P<.001
8W	Column Factor	F (1, 42) = 1.698	P=.200	Column Factor	F (1, 41) = 0.057	P=.813
**VPM T2 mEPSC**	ANOVA table	F (DFn, DFd)	P value	ANOVA table	F (DFn, DFd)	P value
2W	Interaction	F (2, 37) = 0.315	P=.732	Interaction	F (2, 39) = 1.796	P=.179
4W	Row Factor	F (2, 37) = 3.708	P=.034	Row Factor	F (2, 39) = 9.070	P<.001
8W	Column Factor	F (1, 37) = 2.468	P=.125	Column Factor	F (1, 39) = 0.055	P=.817
**VPL sIPSC**	ANOVA table	F (DFn, DFd)	P value	ANOVA table	F (DFn, DFd)	P value
2W	Interaction	F (2, 51) = 5.154	P=.009	Interaction	F (2, 50) = 0.636	P=.534
4W	Row Factor	F (2, 51) = 1.746	P=.185	Row Factor	F (2, 50) = 2.36	P=.105
8W	Column Factor	F (1, 51) = 8.692	P=.005	Column Factor	F (1, 50) = 0.179	P=.674
**VPM sIPSC**	ANOVA table	F (DFn, DFd)	P value	ANOVA table	F (DFn, DFd)	P value
2W	Interaction	F (2, 47) = 2.459	P=.096	Interaction	F (2, 46) = 0.8210	P=.446
4W	Row Factor	F (2, 47) = 0.8082	P=.452	Row Factor	F (2, 46) = 2.474	P=.095
8W	Column Factor	F (1, 47) = 9.487	P=.003	Column Factor	F (1, 46) = 2.707	P=.107
**VPL mIPSC**	ANOVA table	F (DFn, DFd)	P value	ANOVA table	F (DFn, DFd)	P value
2W	Interaction	F (2, 58) = 2.017	P=.142	Interaction	F (2, 60) = 0.154	P=.858
4W	Row Factor	F (2, 58) = 2.523	P=.089	Row Factor	F (2, 60) = 5.093	P=.009
8W	Column Factor	F (1, 58) = 11.45	P=.001	Column Factor	F (1, 60) = 1.171	P=.284
**VPM mIPSC**	ANOVA table	F (DFn, DFd)	P value	ANOVA table	F (DFn, DFd)	P value
2W	Interaction	F (2, 47) = 0.008	P=.992	Interaction	F (2, 54) = 0.390	P=.679
4W	Row Factor	F (2, 47) = 1.750	P=.185	Row Factor	F (2, 54) = 11.8	P<.001
8W	Column Factor	F (1, 47) = 5.310	P=.026	Column Factor	F (1, 54) = 1.17	P=.284

Supplemental Table 4. Summary of imaging data and statistics.

	Raw data: Mean (SD)						D'Agostino-Pearson test		Spearman's test		ANOVA table		
	WT			DS			WT	DS	WT	DS			
**VPL # total**	M	SD	n	M	SD	n	K2	p-value	R	p-value	ANOVA table	F (DFn, DFd)	p-value
2W	943	189	8	908	133	8					Interaction	(2, 42) = 0.6	P=.547
4W	1706	216	8	1498	277	8	0.48	0.788	0.17	0.119	Age	(2, 42) = 44.	P<.001
8W	1637	275	8	1568	280	8					Genotype	(1, 42) = 2.3	P=.131
**VPM # total**	M	SD	n	M	SD	n	K2	p-value	R	p-value	ANOVA table	F (DFn, DFd)	p-value
2W	995	172	8	923	154	8					Interaction	2, 42) = 0.09	P=.914
4W	1535	199	8	1417	210	8	5.75	0.056	0.18	0.110	Row Factor	(2, 42) = 28.	P<.001
8W	1396	258	8	1267	201	8					Column Factor	(1, 42) = 3.3	P=.074
**VPL # VGLUT1**	M	SD	n	M	SD	n	K2	p-value	R	p-value	ANOVA table	F (DFn, DFd)	p-value
2W	907	217	7	1042	197	7					Interaction	(2, 40) = 1.3	P=.270
4W	1772	311	8	1631	312	8	0.43	0.806	0.17	0.135	Row Factor	(2, 40) = 33.	P<.001
8W	1637	170	8	1508	281	8					Column Factor	1, 40) = 0.35	P=.556
**VPM # VGLUT1**	M	SD	n	M	SD	n	K2	p-value	R	p-value	ANOVA table	F (DFn, DFd)	p-value
2W	785	176	7	1069	145	7					Interaction	(2, 39) = 1.9	P=.163
4W	1346	286	7	1327	283	8	0.32	0.852	-0.10	0.257	Row Factor	(2, 39) = 22.	P<.001
8W	1335	187	8	1479	119	8					Column Factor	(1, 39) = 4.8	P=.035
**VPL # VGLUT2**	M	SD	n	M	SD	n	K2	p-value	R	p-value	ANOVA table	F (DFn, DFd)	p-value
2W	148	30	7	135	30	7					Interaction	(2, 40) = 3.3	P=.045
4W	187	34	8	123	25	8	2.79	0.248	0.19	0.099	Row Factor	(2, 40) = 0.7	P=.500
8W	191	46	8	117	35	8					Column Factor	(1, 40) = 25.	P<.001
**VPM # VGLUT2**	M	SD	n	M	SD	n	K2	p-value	R	p-value	ANOVA table	F (DFn, DFd)	p-value
2W	97	30	7	113	27	7					Interaction	(2, 39) = 0.7	P=.485
4W	108	16	8	101	17	8	0.25	0.882	0.08	0.300	Row Factor	(2, 39) = 0.1	P=.833
8W	107	29	7	112	27	8					Column Factor	(1, 39) = 0.4	P=.498
